# The Conserved Tyr176/Leu177 Motif in the α-Helix 9 of the Feline Immunodeficiency Virus Capsid Protein Is Critical for Gag Particle Assembly

**DOI:** 10.3390/v11090816

**Published:** 2019-09-04

**Authors:** César A. Ovejero, Silvia A. González, José L. Affranchino

**Affiliations:** Laboratorio de Virología, Consejo Nacional de Investigaciones Científicas y Técnicas (CONICET)-Universidad de Belgrano (UB), Villanueva 1324, Buenos Aires C1426BMJ, Argentina (C.A.O.) (S.A.G.)

**Keywords:** feline immunodeficiency virus, Gag polyprotein, capsid protein, retrovirus assembly, retrovirus infectivity

## Abstract

The capsid domain (CA) of the lentiviral Gag polyproteins has two distinct roles during virion morphogenesis. As a domain of Gag, it mediates the Gag–Gag interactions that drive immature particle assembly, whereas as a mature protein, it self-assembles into the conical core of the mature virion. Lentiviral CA proteins are composed of an N-terminal region with seven α-helices and a C-terminal domain (CA-CTD) formed by four α-helices. Structural studies performed in HIV-1 indicate that the CA-CTD helix 9 establishes homodimeric interactions that contribute to the formation of the hexameric Gag lattice in immature virions. Interestingly, the mature CA core also shows inter-hexameric associations involving helix 9 residues W184 and M185. The CA proteins of feline immunodeficiency virus (FIV) and equine infectious anemia virus (EIAV) exhibit, at equivalent positions in helix 9, the motifs Y176/L177 and L169/F170, respectively. In this paper, we investigated the relevance of the Y176/L177 motif for FIV assembly by introducing a series of amino acid substitutions into this sequence and studying their effect on in vivo and in vitro Gag assembly, CA oligomerization, mature virion production, and viral infectivity. Our results demonstrate that the Y176/L177 motif in FIV CA helix 9 is essential for Gag assembly and CA oligomerization. Notably, mutations converting the FIV CA Y176/L177 motif into the HIV-1 WM and EIAV FL sequences allow substantial particle production and viral replication in feline cells.

## 1. Introduction

Feline immunodeficiency virus (FIV), like all other lentiviruses, assembles at the plasma membrane of the infected cells [1]. The viral Gag polyprotein multimerizes into immature virions that bud into the extracellular medium [1]. In this regard, we have previously demonstrated that the FIV Gag precursor, when expressed in mammalian cells in the absence of other viral proteins, assembles into virus-like particles (VLPs) morphologically similar to immature virions [2]. In addition, we have shown that the recombinant FIV Gag protein produced in *Escherichia coli* is capable of self-assembling in vitro into 33-nm spherical particles [3]. Likewise, the Gag polyproteins of human and simian immunodeficiency viruses (HIV-1 and SIV, respectively) can assemble into VLPs both in vivo and in vitro [4,5,6,7,8,9,10]. Taken together, these observations underscore the notion that lentiviral Gag polyproteins contain all the molecular determinants necessary for virion assembly and budding [11,12].

Concomitantly with virus budding from the host cell, the FIV Gag precursor is cleaved by the virus-encoded protease into its functional domains: matrix (MA), capsid (CA), spacer peptide p1, nucleocapsid (NC), and the C-terminal p2 peptide [13]. Lentiviral Gag processing causes a series of structural rearrangements that transforms the spherical Gag shell of the immature virion into the mature infectious particle, which exhibits a layer of the MA protein directly underneath the cell-derived viral membrane and the characteristic lentiviral conical core formed by the CA polypeptide [11,12]. Of note, the central CA domain of Gag plays distinct roles during lentiviral morphogenesis. As part of the Gag precursor, the CA establishes the Gag self-interactions that drive the assembly of HIV-1, SIV, and FIV immature virions [10,14,15], which exhibit an array of Gag molecules in an hexagonal lattice, as has been demonstrated for HIV-1 [16,17,18,19,20]. As an independent protein of the mature virion, the CA self-assembles into the core structure [21,22,23], which protects the viral components such as the NC–genomic RNA complex, reverse transcriptase, and integrase, which are essential for virus infection and spreading [24,25]. Notably, it has been shown that the HIV-1 core is formed by hexamers of the CA together with 12 pentamers that allow curvature and closure of the cone-shaped core [26].

Interestingly, the functions of the CA are not limited to lentiviral assembly. The mature CA has been shown to be also involved in virion uncoating and in nuclear import of the preintegration complex to the cell nucleus [27,28,29]. In addition, it has been reported that positively charged pores in the HIV-1 CA allow the recruitment of nucleotides into the capsid interior [30].

Lentiviral CA proteins are organized in two α-helical regions: an N-terminal domain (CA-NTD, helices 1–7) and a C-terminal domain (CA-CTD, helices 8–11) connected by a short flexible hinge [21,31,32,33]. The CA-CTD exhibits a 20-amino-acid motif, known as the major homology region (MHR), which is critical for Gag particle assembly [10,15,34,35].

Several structural studies on the HIV-1 CA have revealed that the hexagonal Gag lattice of the immature virions is maintained by extensive inter- and intra-hexameric CA-NTD contacts, whereas the CA-CTD forms inter-hexameric homodimers [16,17,18,19,20]. In addition, the C-terminus of the HIV-1 CA-CTD together with the N-terminal 8 residues of the spacer peptide SP1 forms a six-helix bundle that helps stabilize the immature Gag lattice [36,37].

Processing of Gag by the viral protease into its distinct individual domains leads to the assembly of the mature core. In each mature HIV-1 CA hexamer, helices 1–3 of the CA-NTDs form a central 18-helix bundle, whereas the CA-CTDs engage in dimeric inter-hexamer interactions mediated mainly by the hydrophobic residues W184 and M185 in helix 9 [21,23,26,38,39,40,41]. Of note, when comparing the CA–CA interactions that mediate immature particle assembly with those found in the core of mature virions, the CA-CTD forms, in both cases, homodimeric inter-hexamer interactions involving W184 and M185 in helix 9. Therefore, helix 9 of the lentiviral CA-CTD appears to be important for both immature virion assembly and mature core formation.

In FIV, we have obtained several lines of evidence highlighting the relevance of the CA protein and, in particular its CTD, for Gag assembly: (i) among a series of FIV Gag subdomains, the CA–p1–NC region associates with wild-type Gag with the highest efficiency [1,15]; (ii) a chimeric SIV carrying the FIV CA-CTD assembles into virions as efficiently as the wild-type virus [1,42]; and (iii) the FIV CA-CTD dimerizes in vitro and forms high-molecular-weight oligomers [1,42].

In this paper, we analyzed the role that the FIV CA-CTD plays in the formation of immature and mature virions by introducing a series of amino acid substitutions affecting the highly conserved Y176/L177 motif in the CA helix 9.

## 2. Materials and Methods

### 2.1. Cell Lines

The COS-7 African green monkey kidney and Crandell feline kidney (CrFK) cell lines, obtained from the American Type Culture Collection (ATCC, USA), were cultured at 37 °C in Dulbecco’s modified Eagle’s medium (DMEM, GIBCO, Carlsbad, CA, USA) supplemented with 10% fetal bovine serum (GIBCO).

### 2.2. DNA Constructs and Site-Directed Mutagenesis

The FIV constructs were derived from the infectious molecular clone FIV-14 of the Petaluma isolate [43]. Mutations in the *gag* region encoding the CA domain were introduced by asymmetric PCR-based site-directed mutagenesis using the Q5 High-Fidelity DNA polymerase (New England BioLabs, Ipswich, MA, USA) and antisense oligonucleotides carrying the desired mutations, as we have previously described [44]. The single or double mutations affecting the CA residues at positions 176 and 177 were introduced into a SacI-NcoI restriction fragment corresponding to nucleotides (nt) 508–2499 of the FIV genome. HindIII-EcoRI (nt 1242–1872) fragments containing the corresponding mutations were substituted for the wild-type counterpart in the pcDNA-FIV *gag* plasmid coding for wild-type FIV Gag (nt 628–1980 of the FIV-14 genome) [15]. To generate the proviral FIV DNA constructs encoding the amino acid substitutions Y176F or Y176W/L177M in the CA domain, the SacI-NcoI restriction fragments (nt 508–2499) carrying these mutations were used to replace the equivalent wild-type region in the context of the FIV genome cloned in the pSV-SPORT1 plasmid [45]. All DNA constructs were completely sequenced to verify the absence of fortuitous mutations (Macrogen Latin America, Buenos Aires, Argentina).

### 2.3. Sequence Similarity Searching

The amino acid sequence of FIV-14 CA helix 9 (15 residues) was used as a query to search for sequence similarity against databases of non-redundant protein and translated nucleotide sequences assisted by the Protein BLAST and TBLASTN programs, respectively, (https://blast.ncbi.nlm.nih.gov/Blast.cgi).

### 2.4. Transfections and Viral Protein Analysis

Expression of the wild-type and mutant FIV *gag* genes was performed using the vaccinia T7 system essentially as previously described [10,15]. Briefly, confluent monolayers of COS-7 cells (35-mm-diameter dishes) were infected with the vT7-3 recombinant vaccinia virus expressing the T7 RNA polymerase (kindly provided by Dr. B. Moss, NIAID, NIH, USA) at a multiplicity of 10 for 1 h at 37 °C. After infection, cells were washed twice with DMEM, and transfected with 2.5 μg of the plasmid constructs using Lipofectamine 3000 (Invitrogen-Thermo Fisher Scientific, Waltham, MA, USA). Thirty hours post-transfection, cells were harvested and lysed at 4 °C in a buffer containing 50 mM Tris-HCl (pH 8.0), 150 mM NaCl, 1% Nonidet P-40, and Protease Inhibitor Cocktail (Roche, Basel, Switzerland). The culture supernatants from the infected/transfected cells were clarified by filtration through 0.45-µm pore-size syringe filters, and VLPs were purified from the supernatants by ultracentrifugation (100,000× *g*, 90 min, 4 °C) through a 20% (*w*/*v* in phosphate-buffered saline [PBS]) sucrose cushion, as we have previously described [10,42]. Cell- and VLPs-associated viral proteins were resolved by sodium dodecyl sulfate (SDS)-polyacrylamide gel electrophoresis (SDS-PAGE), blotted onto nitrocellulose membranes, and analyzed by Western blotting coupled with an enhanced chemiluminescence assay (Western Lightning ECL Pro, PerkinElmer, Waltham, MA, USA). To detect the wild-type and mutant Gag proteins, we used the anti-FIV CA monoclonal antibody (MAb) PAK3-2C1 that is specific for the CA MHR [42] (NIH AIDS Reagent Program, Division of AIDS, NIAID, NIH), and horseradish peroxidase (HRP)-conjugated anti-mouse immunoglobulin (Cayman Chemical, Ann Arbor, MI, USA) as secondary antibody. Blots were exposed to Amersham Hyperfilm ECL (GE Healthcare Life Sciences, Chicago, IL, USA). Films were scanned as image files using an Epson Perfection V700 Photo/Film scanner (Seiko Epson Corporation, Suwa, Nagano, Japan), and the Gag protein bands in these images were quantified using ImageJ software (https://image.nih.gov/ij/).

For the expression of the wild-type and mutant Y176F and Y176W/L177M FIV Gag polyproteins in the context of the viral genome, CrFK cells (grown in 35-mm-diameter dishes) were transfected with the proviral DNAs, as we have previously described [44,46]. At 48 h post-transfection, cell and virion lysates were obtained as previously reported [44,46], and the FIV Gag and CA proteins were detected by Western blotting, as described above.

### 2.5. Expression in Escherichia coli and Purification of Recombinant Proteins

The FIV *gag* genes carrying the Y176A, Y176W, or L177A mutations were PCR amplified and cloned into the EcoRV and SalI sites of the pET-30b (+) plasmid vector (Novagen, Merck, Darmstadt, Germany). Generation of the pET-FIV*gag* plasmid that directs the expression of wild-type FIV Gag with an N-terminal six-histidine tag, has been described previously [3]. Production in *E. coli* BL21 (DE3) and purification by affinity chromatography of histidine-tagged Gag proteins followed protocols reported previously [3,10]. Protein concentrations were estimated as we have already described [10,47]. Recombinant Gag proteins were stored at −80 °C until further use.

### 2.6. In Vitro Assembly of FIV Gag Proteins

Cloning of the FIV encapsidation signal (nt 216–947 of FIV-14; referred to here as FIV R-U5-MA) in the pGEM-5Zf(+) vector (Promega, Madison, WI, USA) and the in vitro synthesis of the R-U5-MA RNA using the plasmid as template have been reported previously [3,48].

Purified recombinant histidine-tagged wild-type and mutant FIV Gag proteins were thawed on ice and centrifuged at 16,000× *g* for 20 min, and aliquots from the resulting supernatants were then used in the in vitro assembly reactions, as we have described previously [3]. Succinctly, 5 µg of the recombinant Gag proteins were incubated for 3 h at 37 °C in a solution containing 50 mM Tris-HCl (pH 8.0), 150 mM NaCl, 5 mM dithiothreitol (DTT), 10 µM ZnCl_2_, 20 ng/µL in-vitro-transcribed FIV R-U5-MA RNA, and 4 units/µL recombinant RNasin ribonuclease inhibitor (Promega). The assembly reactions were analyzed by sedimentation assays (16,000× *g*, 60 min, 4 °C) to separate the pelletable assembled structures from the unassembled Gag molecules [3,10,42]. The Gag proteins in the supernatant and pellet fractions were resolved by SDS-PAGE, blotted onto nitrocellulose membranes, and detected by immunoblotting with the MAb PAK3-2C1 specific for the FIV CA MHR. The quantitation of Western blot signals was performed as described above.

### 2.7. In Vitro Assembly of FIV CA Proteins

We have previously reported that the region of the FIV *gag* gene coding for the FIV CA protein (nt 1033–1698 of FIV-14) was PCR amplified and cloned into the SalI-NotI sites of pET-30b(+) so as to express, in *E. coli* strain BL21(DE3), the CA polypeptide with an N-terminal six-histidine tag [42]. Following the same strategy as that for wild-type CA, the His-tagged CA protein containing the Y176A amino acid substitution was expressed in *E. coli* and purified by affinity chromatography. The recombinant wild-type and mutant CA proteins were used in in vitro assembly reactions as we have described previously [42]. Briefly, assembly reactions were performed at a protein concentration of 1 µg/µL in 50 mM Tris-HCl (pH 8.0) buffer containing 1 M NaCl and 5 mM DTT. After 16 h of incubation at 8 °C, the CA-derived assemblies were analyzed by sedimentation assays [42].

### 2.8. Reverse Transcriptase Assays

Quantitative determination of virion-associated reverse transcriptase (RT) was performed using a commercial colorimetric assay kit (Roche), as we have previously described [49,50]. Briefly, viruses from the cell-free culture supernatants of transfected or infected CrFK cells were concentrated by the addition of a half volume of 30% polyethylene glycol 8000 (*w*/*v*) and 1.2 M NaCl. After 16 h at 4 °C, the mixtures were centrifuged at 8000× *g* for 10 min, and the virions were lysed by resuspending the pellet in 50 mM Tris-HCl (pH 7,8), 80 mM KCl, 2.5 mM DTT, 0.75 mM EDTA, and 0.5% Triton X-100. The lysed viral samples were mixed with a buffer containing an optimized ratio of biotin-labeled and digoxigenin-labeled nucleotides and a poly(rA).oligo(dT)_15_ template–primer hybrid and incubated for 2 h at 37 °C. The detection and quantification of synthesized DNA as a parameter for RT activity follows a sandwich ELISA protocol: synthesized biotin-labeled DNA binds to microplate modules precoated with streptavidin, and the DNA is then detected by a HRP-conjugated antibody specific for digoxigenin and 2,2′-Azino-bis(3-ethylbenzthiazoline-6-sulfonic acid) substrate. The reaction produces a colored product whose absorbance is measured on a microtiter plate (ELISA) reader at 405 nm (reference wavelength 490 nm). The absorbance of the samples is correlated to the calibration curve obtained with different amounts of the recombinant HIV-1 RT enzyme provided in the kit. The RT assays were performed in duplicate from at least three independent experiments.

### 2.9. FIV Infectivity Assays in CrFK Cells

To obtain replication kinetics data in feline CrFK cells, we first generated virus stocks by transfection of this cell line with the wild-type, Y176F, and Y176W/L177M proviral DNAs, as described above. CrFK cells were infected with volume-adjusted supernatants containing equivalent RT levels, previously treated with DNase I at 25 °C for 20 min to remove potentially contaminating plasmid DNA. Supernatants were added to 4 × 10^5^ CrFK cells in the presence of 20 µg/mL DEAE-dextran and allowed to adsorb for 4 h. The cells were washed twice with PBS to remove residual virus and incubated with fresh medium. Every 2 days, half of the cell culture medium was harvested and replaced with an equal volume of fresh medium. The aliquots of culture supernatants were clarified by filtration through 0.45-µm-pore-size syringe filters and frozen at −80 °C for RT quantitation at the conclusion of the experiment.

## 3. Results

### 3.1. Mutagenesis of Residues Y176 and L177 in the FIV CA-CTD

It has been demonstrated that HIV-1 first assembles into immature particles composed of a lattice of Gag hexamers [11]. Fitting the high-resolution structure of the HIV-1 CA-CTD into intact immature virions showed that helix 9 forms a homodimeric interface linking neighboring hexamers, and that the CA residues W184 and M185 are critical for this interface interaction [20]. In this regard, the substitution of W184 or M185 by alanine impairs HIV-1 particle assembly [14].

**Figure 1:**
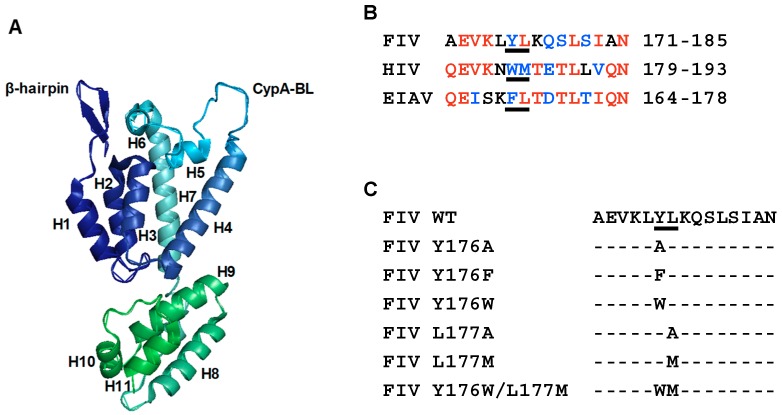
Mutagenesis of the Y176 and L177 residues in helix 9 of the FIV CA protein. (**A**) Ribbon representation of the crystal structure of the FIV CA (PDB 5NA2). The α-helices H1–H7 and H8–H11, corresponding to the N-terminal domain (CA-NTD) and C-terminal domain (CA-CTD), respectively, are shown. The N-terminal β-hairpin and the cyclophilin A-binding loop (CypA-BL) are also indicated. (**B**) Alignment of the amino acid sequences of helix 9 of FIV [33], human immunodeficiency virus type 1 (HIV-1) [51], and equine infectious anemia virus (EIAV) [31] CA proteins. Identical amino acids at the same positions in two or three sequences are highlighted in red, whereas similar residues are indicated in blue. The assembly-relevant motif W184/M185 of HIV-1 CA as well as the amino acids present at equivalent positions in the FIV and EIAV CA helix 9 are underlined. (**C**) Amino acid substitutions that were designed to modify the Y176/L177 motif in the FIV CA. Below the sequence of the wild-type (WT) FIV CA helix 9, the amino acids replacing Y176, L177, or both residues of the motif are listed.

The 1.67 Å crystal structure of the FIV CA has recently been determined (Figure 1A) [33] and, although the linker connecting the CA-NTD and CA-CTD is shorter than those found in the HIV-1 and equine infectious anemia virus (EIAV) CA proteins [31,51], the FIV CA-NTD and CA-CTD can still be individually superposed with those of HIV-1 and EIAV [33]. Therefore, it can be speculated that the FIV CA-CTD and, in particular helix 9, may be involved in CA-mediated Gag multimerization.

Alignment of the amino acid sequences of the helix 9 of FIV, HIV-1, and EIAV CA-CTDs [31,33,51] shows that the HIV-1 CA motif W184/M185 is not present in either of these nonprimate lentiviruses (Figure 1B). Instead, FIV exhibits, at equivalent positions in the CA helix 9, the amino acid sequence Y176/L177, whereas EIAV contains the F169/L170 residues (Figure 1B). Of note, helix 9 motif Y176/L177 is highly conserved among FIV isolates according to Protein BLAST and TBLASTN analyses. Indeed, this motif is present in 183 FIV Gag and CA protein sequences (Figure S1), and in 221 FIV translated Gag-encoding and CA-encoding nucleotide sequences (Figure S2). Therefore, we sought to investigate the relevance of this amino acid sequence for FIV Gag particle assembly by first introducing into the CA domain a series of single amino acid substitutions and a double mutation (Figure 1C), and then characterizing the effect of these changes in the CA amino acid sequence on FIV particle production and infectivity.

### 3.2. Effect of Mutations Affecting the CA-CTD Motif Y176/L177 on Gag Particle Assembly

To establish whether the Y176/L177 motif of the FIV CA helix 9 has any role in Gag multimerization, we introduced five single amino acid substitutions and a double mutation affecting this CA motif (Figure 1C) in the context of a plasmid construct that directs the synthesis of the FIV Gag polyprotein using the vaccinia T7 system (see Materials and Methods). We chose to express the CA mutants in the context of Gag in the absence of polyprotein processing by the viral protease so as to solely examine their phenotype with respect to immature particle production.

**Figure 2:**
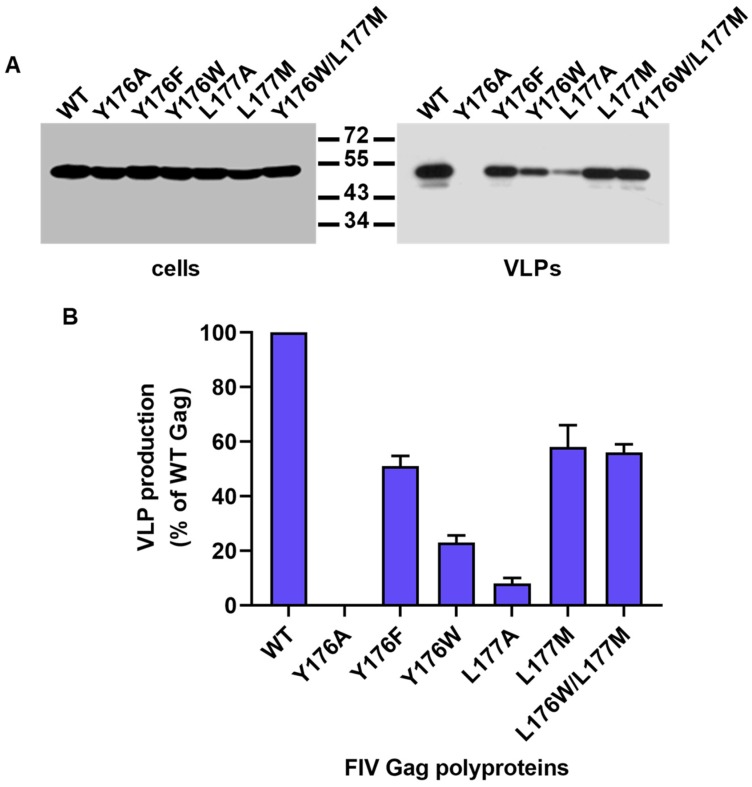
Assembly of FIV Gag mutants into VLPs. (**A**) COS-7 cells were first infected with the vTF7-3 recombinant vaccinia virus, and then transfected with the plasmids directing the expression of wild-type (WT) or the mutant Gag polyproteins. Cells were harvested 30 h post-transfection, and the VLPs were purified from the clarified culture supernatants as described in Materials and Methods. The presence of Gag proteins in cell and VLPs lysates was analyzed by Western blotting using the anti-FIV CA MAb. Numbers indicate the positions of the molecular weight standards (in kDa). The results are representative of three independent experiments. (**B**) The amount of each Gag mutant polyprotein in the VLPs fraction was quantitated and referred to that purified from the clarified supernatant of cells expressing wild-type Gag (considered as 100%). Data presented are the mean of three independent experiments ± the standard deviation.

COS-7 cells expressing the wild-type and mutant Gag proteins were lysed, and the VLPs were purified from the clarified culture supernatants as described in Materials and Methods. The Gag proteins were detected in cell and VLPs lysates by Western blot using the anti-FIV CA MAb. Figure 2A shows that all the CA mutants expressed wild-type levels of the Gag protein. Amino acid substitution Y176A abolished particle production, whereas mutations Y176W and L177A impaired VLP assembly (Figure 2A). By contrast, amino acid substitutions Y176F, L177M, and Y176W/L177M allowed substantial Gag assembly into particles (Figure 2A).

Quantitation in VLPs of the relative protein levels of wild-type Gag and the CA mutants revealed that: (i) Gag protein was undetectable in the case of mutant Y176A; (ii) mutants Y176W and L177A produced particles at levels representing 23 ± 3% and 8 ± 3% of wild-type Gag, respectively; and (iii) Gag polyproteins carrying the CA amino acid substitutions Y176F, L177M, or Y176W/L177M assembled into VLPs with efficiencies of 51 ± 4%, 58 ± 8%, and 56 ± 3% with respect to wild-type Gag, respectively (Figure 2B).

### 3.3. In Vitro Assembly Phenotype of the Gag Polyproteins Carrying the Y176A, Y176W, and L177A CA Mutations

We have previously demonstrated that the full-length FIV Gag protein expressed in *E. coli* and purified by affinity chromatography assembles in vitro into spherical particles that are morphologically similar to immature virions [3].

**Figure 3:**
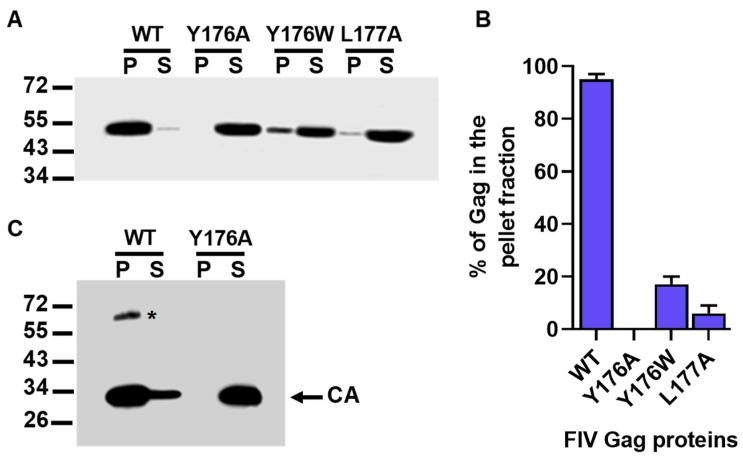
In vitro assembly reactions for the recombinant FIV Gag polyproteins containing the Y176A, Y176W, and L177A amino acid substitutions in the CA domain. In vitro oligomerization ability of the FIV CA Y176A mutant polypeptide. (**A**) Sedimentation analysis of the assembly reactions of purified wild-type (WT) and mutant His-FIV Gag proteins. After incubation of the assembly mixtures, the pellet (P) and supernatant (S) fractions were separated by centrifugation. The P and S fractions were then analyzed for the presence of Gag protein by Western blotting using the anti-FIV CA MAb. Results shown are representative of three independent experiments. (**B**) The percentage of the total amount of each Gag polyprotein that partitions into the P fraction was quantitated. Values represent the mean of three independent experiments ± the standard deviation. (**C**) Sedimentation analysis of the in vitro assembly reactions for recombinant wild-type (WT) and Y176A FIV CA proteins. The purified His-FIV CA polypeptides were incubated under the conditions described in Materials and Methods, and the partitioning of the His-FIV CA proteins into the pellet (P) and supernatant (S) fractions was determined by Western blotting using the MAb specific for the FIV CA. The asterisk (*) indicates SDS-resistant CA dimers present in the P fraction of the wild-type CA protein.

The data described in the previous section indicated that the Y176A mutant Gag polyprotein, when expressed in COS-7 cells, was defective in particle assembly, whereas amino acid substitutions Y176W and L177A reduced VLPs production by 77% and 92%, respectively. To determine whether these severe impairments in particle formation were caused by a direct effect of the mutations on Gag self-assembly and not on other processes such as Gag transport to the cell surface or stable Gag association with the plasma membrane, we tested the capacity of the Y176A, Y176W, and L177A Gag proteins to assemble in vitro. To this end, wild-type FIV Gag and the mutant Gag polyproteins Y176A, Y176W, and L177A were expressed in *E. coli*, and the purified histidine-tagged proteins were incubated in parallel at 37 °C for 3 h, as described in Materials and Methods. Then, the assembly reactions were centrifuged to separate the pelletable Gag particles from the unassembled Gag molecules that remain in the supernatant. The analysis by Western blotting of the resulting pellet (P) and supernatant (S) fractions showed that, as expected, most of the wild-type FIV Gag protein (95 ± 2%) was found in the P fraction (Figure 3A,B). By contrast, the FIV Gag precursor carrying the Y176A amino acid substitution in the CA domain partitioned to the S fraction (Figure 3A,B). In the case of the recombinant Gag polyprotein containing the CA mutation Y176W, only 17 ± 3% of the total Gag protein was detected in the P fraction, whereas as little as 6 ± 3% of the total amount of the L177A Gag mutant partitioned into the pellet after centrifugation of the assembly reaction (Figure 3A,B). These results confirm that the single amino acid replacements Y176A, Y176W, and L177A in the FIV CA cause a severe defect in the ability of the Gag polyprotein to self-assemble into multimeric complexes.

### 3.4. Oligomerization Ability of the Y176A FIV CA Mutant

We have previously reported that the FIV CA expressed in *E. coli* as an N-terminally His-tagged polypeptide and purified by affinity chromatography is capable of oligomerizing in vitro [42]. Since the single amino acid substitution Y176A in the helix 9 of the FIV CA is sufficient to abrogate Gag particle assembly, we decided to study the effect of this mutation on recombinant CA oligomerization.

Both the wild-type and Y176A FIV CA polypeptides were expressed in *E. coli* as His-tagged fusion proteins and purified by affinity chromatography. Then, the recombinant CA proteins were tested for their ability to oligomerize, as described in Materials and Methods, and the products of the in vitro assembly reactions were analyzed by sedimentation assays, as we have previously described [42]. As expected according to our previous results [42], analysis of the P and S fractions obtained after centrifugation of the assembly reactions showed that a major proportion of the wild-type FIV CA protein partitioned to the P fraction (Figure 3C). In addition, an SDS-resistant protein species that represents CA dimers was detected in the P fraction (Figure 3C) [42]. In this regard, we have also found that the SIV CA protein is also capable of forming SDS-resistant dimers in vitro [10]. When the P and S fractions resulting from the in vitro assembly reaction of mutant Y176A CA protein were analyzed, we observed that this protein solely partitioned into the S fraction (Figure 3C). This result demonstrates that mutation Y176A prevents multimerization of the mature FIV CA, which is a property that is essential for the formation of the mature virion core [20,26].

### 3.5. Assembly and Infectivity Phenotypes of Proviral DNAs Carrying the Y176F or the Y176W/L177M CA Mutation

Amino acid substitutions Y176F and Y176W/L177M were designed to convert the Y176/L177 motif in the helix 9 of the FIV CA into the sequences F169/L170 and W184/M185 present at equivalent positions in the CA of EIAV and HIV-1, respectively (Figure 1B). Interestingly, our experiments to this point indicated that the Gag polyproteins containing mutations Y176F and Y176W/L177M in the CA domain assemble into VLPs with an efficiency of about 50% with respect to that of wild-type Gag (Figure 2). Based on these results, we decided to introduce individually these mutations into the FIV proviral DNA and investigate in feline cells their effect on virion assembly and virus infectivity.

**Figure 4:**
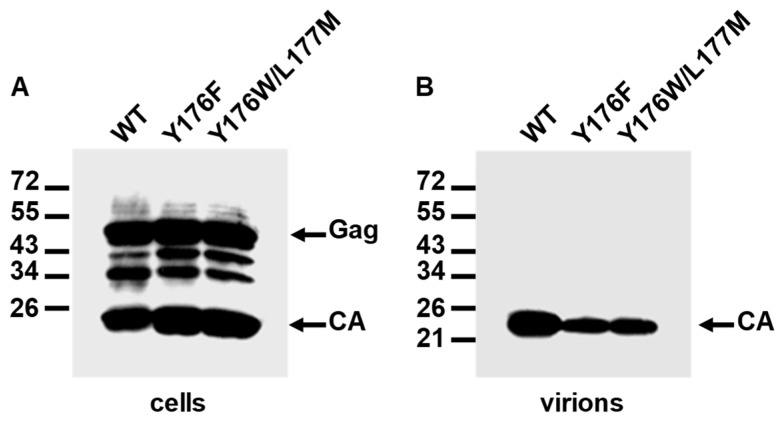

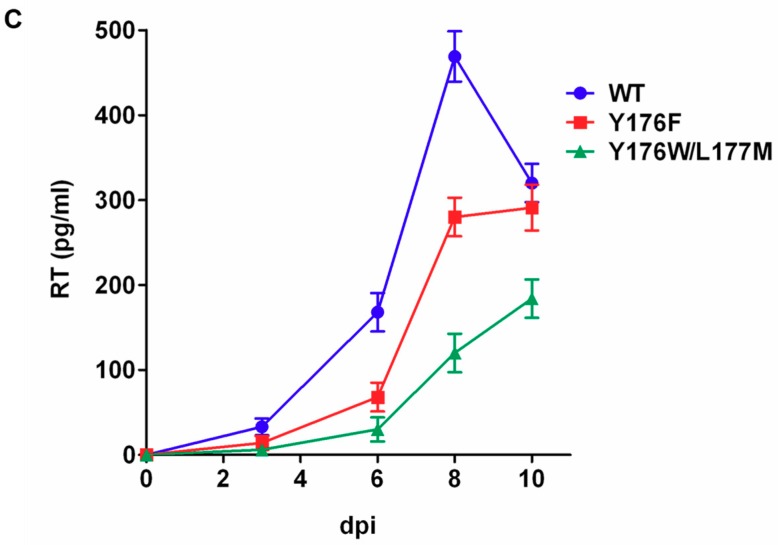
Effect of the Y176F and Y176W/L177M mutations in the FIV CA domain on Gag expression, processing, virion assembly, and virus infectivity. Crandell feline kidney (CrFK) cells were transfected with wild-type FIV-14 (WT) or the CA mutant proviral clones. Viral proteins present in the cell lysates (**A**) or virions (**B**) were detected by Western blot using the MAb specific for the FIV CA protein. The mobilities of the Gag precursor and CA protein are shown, as are the positions of the molecular weight standards (in kDa). (**C**) Replication kinetics of the Y176F and Y176W/L177M CA mutant viruses in CrFK cells. Virus stocks, obtained by the transfection of CrFK cells, were normalized for reverse transcriptase (RT) levels and used to infect CrFK cell monolayers. Virus replication was assessed by measuring RT activity at two-day intervals post-infection. Dpi, days post-infection. The replication curves show the means ± standard deviations from three independent experiments.

Wild-type and mutant proviral DNAs were transfected into feline CrFK cells as described in Materials and Methods. Analysis of the cell and virion lysates by Western blotting using the MAb directed against the FIV CA showed that the Gag polyproteins containing the Y176F and the Y176W/L177M CA mutations were expressed and processed at wild-type levels, and that they assembled into virions (Figure 4A,B). However, quantitation of the amounts of virion-associated CA in three independent experiments showed that mutants Y176F and Y176W/L177M produced extracellular virions in a proportion representing 46 ± 2% and 51 ± 3% of the wild-type proviral DNA, respectively.

We next examined the ability of Y176F and Y176W/L177M CA mutant viruses to replicate in feline CrFK cells. It should be reminded that this feline cell line expresses the FIV coreceptor CXCR4 and that the Petaluma isolate, used in this and other studies of our laboratory, only requires CXCR4 for cell entry [45,46]. CrFK cells were infected with the supernatants of CrFK cells transfected with the FIV-14 or the CA mutant proviral DNAs, and virus replication was monitored over time by measuring RT levels in the cell-free culture supernatants. As shown in Figure 4C, the Y176F and Y176W/L177M viruses were capable of replicating in CrFK cells, although with slower kinetics and attaining lower maximal virus titers than those of the wild-type virus. Of note, the Y176W/L177M mutant virus exhibited throughout the experiment lower levels of RT than those attained by the Y176F virus (Figure 4C). Mock-infected cell cultures were consistently negative for RT activity. For the sake of clarity, these data were not included in Figure 4C.

## 4. Discussion

In HIV-1, it has been shown that a homodimeric interface formed by the hydrophobic residues W184 and M185 of CA helix 9 contributes to the assembly of both immature virions and mature cores [26]. At equivalent positions in helix 9, the FIV CA exhibits the highly conserved Y176/L177 motif [33]. Taking into account that we have previously shown that the FIV CA-CTD can dimerize in vitro [42], we investigated whether residues Y176 and/or L177 play any role in Gag assembly and CA oligomerization.

When the assembly phenotype of a series of Gag polyproteins carrying amino acid substitutions affecting the FIV CA Y176/L177 motif was analyzed, we found that replacement of Y176 by alanine abrogated the formation of VLPs. Moreover, mutations Y176W and L177A drastically reduced particle assembly by 80% and 90% with respect to wild-type Gag, respectively. In vitro assembly assays performed with recombinant Gag proteins carrying mutations Y176A, Y176W, and L177A demonstrated that these amino acid substitutions have a direct effect on the ability of Gag to multimerize into particles. Taken together, these results indicate that FIV CA residues Y176 and L177 are critical for immature particle assembly. Of note, the recombinant mutant Y176A CA protein was found to be incapable of oligomerizing in vitro, which suggests that residue Y176 contributes to the CA self-interactions that drive mature core formation. Recently, Reed et al. [52] reported that FIV Gag forms complexes similar to HIV-1 capsid assembly intermediates derived from host RNA granules, and that it associates with the host factors ATP-binding cassette protein E1 (ABCE1), DEAD-box helicase 6 (DDX6), and mRNA Decapping protein 2 (DCP2). Four FIV Gag mutants that appeared to be arrested at these assembly intermediates were defective in VLP assembly [52]. Interestingly, one of these mutants was a Gag polyprotein carrying the double amino acid substitution Y176A/L177A in the CA domain [52]. This is in agreement with the assembly-defective phenotype that we observed for mutants Y176A and L177A.

An interesting result stemming from our work is the demonstration that CA mutants Y176F and Y176W/L177M, when expressed in the context of either a construct directing the synthesis of the FIV Gag polyprotein or the viral genome, assemble into particles with an efficiency of about 50% of that of their wild-type counterpart. These amino acid substitutions convert the FIV CA Y176/L177 motif into the FL and WM amino acid sequences present at equivalent positions in the EIAV and HIV-1 CA helix 9, respectively. This indicates that the replacement of FIV CA residues Y176/L177 by WM or FL allows the FIV CA-mediated Gag–Gag interactions that lead to particle assembly to occur with considerable efficiency.

Remarkably, both Y176F and Y176W/L177M mutant viruses are capable of replicating in feline CrFK cells, albeit with slower kinetics and lower maximum titers than those of wild-type FIV. Moreover, the Y176F virus exhibits a replicative advantage over the Y176W/L177M virus and a significant replication competence when compared with the wild-type virus. That Y176F and Y176W/L177M mutant viruses do not replicate with the same efficiency as that of wild-type FIV may be partially explained by the fact that these mutations reduce virus production by 50%. However, since the Y176W/L177M virus is less replication-competent than the Y176F virus, other steps of the virus life cycle may be impaired in the double mutant virus, such as virus uncoating or the CA-mediated transport of the viral DNA to the nucleus.

In summary, in this study, we characterized the role of the conserved Y176/L177 motif of the FIV CA-CTD in Gag assembly, CA oligomerization, and viral infectivity. Our results suggest that this motif in the FIV CA helix 9 is functionally equivalent to its HIV-1 counterpart, the W184/M185 amino acid sequence, with respect to mediating the CA contacts that are necessary for the assembly of both immature particles and mature viral cores.

## Supplementary Materials

The following are available online at https://www.mdpi.com/1999-4915/11/9/816/s1, Figure S1: Alignment of the amino acid sequence of FIV-14 CA helix 9 with non-redundant FIV Gag and CA protein sequences database (complete and partial sequences) using the BLASTP program; Figure S2: Alignment of the amino acid sequence of FIV-14 CA helix 9 with non-redundant translated FIV *gag* and CA nucleotide sequences database (complete and partial sequences) using the TBLASTN program.
