# Supplementary material for: The Conserved Tyr176/Leu177 Motif in the α-Helix 9 of the Feline Immunodeficiency Virus Capsid Protein Is Critical for Gag Particle Assembly
Source: Viruses. 2019 Sep 4;11(9):816. doi: 10.3390/v11090816 (PMC6783973; doi:10.3390/v11090816)
Supplement: Supplementary file 1 [file viruses-11-00816-s001.pdf]

Supplementary Materials

Figure S1. Alignment of the amino acid sequence of FIV-14 CA helix 9 with non-redundant FIV Gag and CA protein sequences database (complete and partial sequences) using the BLASTP program (protein-protein BLAST; https://blast.ncbi.nlm.nih.gov/Blast.cgi). The query sequence corresponds to residues 171-185 of the FIV-14 CA protein. The GenBank accession number of each sequence included in the alignment is indicated in blue. Dots denote identical amino acids.

Query (CA H9)	1	AEVKLYLKQSLSIAN	15
ABU49231.1	306	....	320
P16087.1	306	....	320
AAK27724.1	306	....	320
Q05313.1	306	....	320
CAA40317.1	306	....	320
ABD16377.1	306	....	320
P19027.1	306	....	320
NP_040972.1	306	....	320
ACX47905.1	306	....	320
AAU12277.1	306	....	320
AAB09309.1	306	....	320
P31821.1	306	....	320
CAC24813.1	306	....	320
CAC24816.1	306	....	320
ABV49614.1	306	....	320
CAC24814.1	306	....	320
AAT99669.1	306	....	320
ABD16370.1	306	....	320
AWA45279.1	306	....	320
ASU11154.1	306	....	320
ABD16375.1	306	....	320
ACV53570.1	306	....	320
ABD16372.1	306	....	320
ABD16371.1	306	....	320
ABD16376.1	306	....	320
BAA07064.1	222	....	236
BAA07069.1	222	....	236
BAA07065.1	222	....	236
BAA07066.1	222	....	236
BAA07068.1	222	....	236
AJD38750.1	195	....	209
AJD38747.1	195	....	209
AJD38746.1	195	....	209
QBA31038.1	206	....	220
QBA31018.1	206	....	220
QBA31035.1	206	....	220
QBA31033.1	206	....	220

QBA31030.1	206	....	220
QBA31031.1	206	....	220
AAN52128.1	171	....	185
AAN52131.1	173	....	187
AAN52127.1	170	....	184
AAN52130.1	163	....	177
AAN52132.1	165	....	179
CAA74181.1	171	....	185
CAA74180.1	171	....	185
AQX36296.1	164	....	178
AQX36285.1	164	....	178
AQX36293.1	164	....	178
AQX36303.1	164	....	178
AQX36291.1	164	....	178
AQX36304.1	164	....	178
AQX36301.1	164	....	178
QCL09064.1	157	....	171
AAN31706.1	155	....	169
ACT85909.1	155	....	169
AAN31686.1	155	....	169
AAN31703.1	155	....	169
AAN31705.1	155	....	169
ACT85893.1	155	....	169
ACT85906.1	155	....	169
AAN31712.1	155	....	169
AAN31695.1	155	....	169
ACT85882.1	155	....	169
ACT85905.1	155	....	169
ACT85920.1	155	....	169
ACT85877.1	155	....	169
AAN31685.1	155	....	169
AAN31690.1	155	....	169
ACT85878.1	155	....	169
AAN31693.1	155	....	169
ACT85892.1	155	....	169
AEK32459.1	155	....	169
ACT85870.1	155	....	169
ACT85898.1	155	....	169
AAN31708.1	155	....	169
ACT85874.1	155	....	169
ACT85913.1	155	....	169
ACT85876.1	155	....	169
ACT85871.1	155	....	169
AAN31696.1	155	....	169
AAN31684.1	155	....	169
ANN44238.1	154	....	168
ANN44244.1	154	....	168
ANN44245.1	154	....	168
ANN44239.1	154	....	168

```
ANN44246.1    154  ...............  168
ANN44242.1    154  ...............  168
ANN44237.1    154  ...............  168
ABX25834.1    327   ..............  340
CAA48157.1    306  ...........N...  320
BAA07067.1    223   ..............  236
AJD38745.1    196   ..............  209
BAA07070.1    222  ........H......  236
ABD16373.1    306  ............M..  320
ACV53568.1    306  ............M..  320
AJD38749.1    195  ............M..  209
AJD38748.1    195  ............M..  209
AAN31709.1    155  ............M..  169
ACT85902.1    155  ............M..  169
ANN44241.1    154  ............M..  168
ACV53569.1    306  .K.............  320
ABD48752.1    141  .............    153
ABD48758.1    141  .............    153
ABD48754.1    141  .............    153
ABD48759.1    141  .............    153
ABD48768.1    141  .............    153
ABD48753.1    141  .............    153
ABD48769.1    141  .............    153
ABD48773.1    141  .............    153
ABD48765.1    141  .............    153
ABD48757.1    141  .............    153
ABD48756.1    141  .............    153
ABX25829.1    331   ...........L..  344
ABO16618.1    331   ...........L..  344
ACY25087.1    306  ....I.......M..  320
ACT85930.1    155  ....I.......L..  169
AEK32460.1    155  ....I.......M..  169
ACT85923.1    155  ....V.......M..  169
ABO16619.1    318   ..R.........T.  331
ABD48763.1    141  ............     152
AAB49922.1    327   ...I.......L..  340
AAT12493.1    306  ....T.......L..  320
AAM13443.1    306  ....T.......L..  320
ABD48767.1    141  .......Q.....    153
AHZ63255.1    307   ..RD........S.  320
AHZ63251.1    307   ..RD........S.  320
AHZ63263.1    307   ..RD........S.  320
AHZ63328.1    307   ..RD........S.  320
AHZ63324.1    307   ..RD........S.  320
AHZ63259.1    307   ..RD........S.  320
AHZ63275.1    307   ..RD........S.  320
AHZ63320.1    307   ..RD........S.  320
AHZ63235.1    307   ..RD........S.  320
AHZ63307.1    307   ..RD........S.  320
```

AHZ63227.1	307	..RD........S.	320
AHZ63239.1	307	..RD........S.	320
AHZ63247.1	307	..RD........S.	320
AHZ63231.1	307	..RD........S.	320
AHZ63334.1	307	..RD........S.	320
AHZ63271.1	307	..RD........S.	320
AHZ63267.1	307	..RD........S.	320
AHZ63336.1	307	..RD........S.	320
AHZ63340.1	307	..RD........S.	320
YP_009507790.1	307	..RD........S.	320
AHZ63283.1	307	..RD........S.	320
AHZ63287.1	307	..RD........S.	320
AHZ63295.1	307	..RD........S.	320
AHZ63291.1	307	..RD........S.	320
AHZ63243.1	307	..RD........S.	320
AHZ63299.1	307	..RD........S.	320
AHZ63303.1	307	..RD........S.	320
AHZ63279.1	307	..RD........S.	320
ABO16611.1	306	D..Q...D....S.	319
ABO16612.1	306	D..Q...D....S.	319
ABO16605.1	306	D..Q...D....S.	319
ABO16616.1	306	D..Q...D....S.	319
AHZ63399.1	306	D..Q...D....S.	319
ABO16614.1	306	D..Q...D....S.	319
ABO16613.1	306	D..Q...D....S.	319
AHZ63424.1	306	D..Q...D....S.	319
ABO16606.1	306	D..Q...D....S.	319
ABO16604.1	306	D..Q...D....S.	319
ABO16615.1	306	D..Q...D....S.	319
ABO16610.1	306	D..Q...D....S.	319
ABO16607.1	306	D..Q...D....S.	319
ABB29306.1	306	D..Q...D....S.	319
AHZ63359.1	306	D..Q...D....S.	319
AHZ63369.1	306	D..Q...D....S.	319
AHZ63389.1	306	D..Q...D....S.	319
AHZ63394.1	306	D..Q...D....S.	319
AHZ63429.1	306	D..Q...D....S.	319
AHZ63354.1	306	D..Q...D....S.	319
AHZ63404.1	306	D..Q...D....S.	319
AHZ63344.1	306	D..Q...D....S.	319
AHZ63434.1	306	D..Q...D....S.	319
ABO16608.1	306	D..Q...D....S.	319
AHZ63349.1	306	D..Q...D....S.	319
AHZ63409.1	306	D..Q...D....S.	319
AHZ63364.1	306	D..Q...D....S.	319
AHZ63419.1	306	D..Q...D....S.	319
AHZ63379.1	306	D..Q...D....S.	319
ABO16609.1	306	D..Q...D....S.	319

Figure S2. Alignment of the amino acid sequence of FIV-14 CA helix 9 with non-redundant translated FIV *gag* and CA nucleotide sequences database (complete and partial sequences) using the TBLASTN program (protein-translated nucleotide sequences; https://blast.ncbi.nlm.nih.gov/Blast.cgi). The query sequence corresponds to residues 171-185 of the FIV-14 CA protein. The GenBank accession number of each sequence included in the alignment is indicated in blue. Dots denote identical amino acids.

Query(CA H9)	1	AEVKLYLKQSLSIAN	15
DQ365596.1	916	....	960
MF370550.1	1168	....	1212
U11820.1	1549	....	1593
EU130945.1	916	....	960
MH325062.1	616	....	660
MH325057.1	616	....	660
AJ304962.1	936	....	980
M59418.1	1549	....	1593
DQ365589.1	916	....	960
L06136.1	916	....	960
DQ365595.1	916	....	960
M36968.1	1543	....	1587
AJ304959.1	936	....	980
DQ365594.1	916	....	960
AJ304960.1	936	....	980
DQ365591.1	916	....	960
GQ406242.1	916	....	960
AY679785.1	916	....	960
GQ422127.1	916	....	960
AY684181.1	916	....	960
AY139111.1	493	....	537
M25381.1	1543	....	1587
AF361320.1	916	....	960
X57002.1	1548	....	1592
MF352016.1	1582	....	1626
AJ304961.1	936	....	980
DQ365590.1	916	....	960
AY139109.1	519	....	563
AY139110.1	494	....	538
MK018168.1	470	....	514
MK018167.1	470	....	514
MK018166.1	470	....	514
MK018165.1	470	....	514
MK018164.1	470	....	514
D37819.1	664	....	708
EU025246.1	916	....	960
AY139107.1	509	....	553
AY139105.1	509	....	553

MH325068.1	616	....	660
MH325071.1	616	....	660
EU117992.1	1681	....	1722
D37823.1	664	....	708
MH325064.1	616	....	660
KM880118.1	583	....	627
KY629391.1	490	....	534
KT984669.1	460	....	504
MH325066.1	616	....	660
MH325070.1	616	....	660
MH325069.1	616	....	660
MH325067.1	616	....	660
MH325065.1	616	....	660
MH325061.1	616	....	660
MH325056.1	616	....	660
MH325055.1	616	....	660
MH325054.1	616	....	660
MH325052.1	616	....	660
MH325051.1	616	....	660
AY139112.1	504	....	548
MH325063.1	616	....	660
AY139106.1	513	....	557
KY629401.1	490	....	534
D37820.1	664	....	708
D37818.1	664	....	708
KM880121.1	583	....	627
Y13866.1	511	....	555
D37822.1	664	....	708
KM880117.1	583	....	627
KY629397.1	490	....	534
KY629396.1	490	....	534
KY629393.1	490	....	534
KY629388.1	490	....	534
KY629385.1	490	....	534
KY629384.1	490	....	534
KY629383.1	490	....	534
AY139108.1	488	....	532
Y13867.1	511	....	555
KY629389.1	490	....	534
KT984671.1	460	....	504
KT984670.1	460	....	504
KT984667.1	460	....	504
KT984666.1	460	....	504
KT984664.1	460	....	504
KT984662.1	460	....	504
KY629400.1	490	....	534
KY629399.1	490	....	534
GQ339864.1	463	....	507
GQ339837.1	463	....	507

GQ339806.1	463	....	507
AY196330.1	463	....	507
AF531066.1	463	....	507
AF531065.1	463	....	507
AF531064.1	463	....	507
AF531063.1	463	....	507
AF531062.1	463	....	507
AF531061.1	463	....	507
AF531058.1	463	....	507
AF531056.1	463	....	507
AF531053.1	463	....	507
AF531052.1	463	....	507
AF531051.1	463	....	507
AF531048.1	463	....	507
X68019.1	916	...........N...	960
KT984668.1	460	....	504
GQ339857.1	463	....	507
AF531049.1	463	....	507
AF531050.1	463	....	507
KY629402.1	490	....	534
KY629394.1	490	....	534
AF531057.1	463	....	507
GQ339830.1	463	....	507
GQ339813.1	463	....	507
AF531076.1	463	....	507
GQ422126.1	916	.K.............	960
DQ365592.1	916	............M..	960
KT984663.1	460	....	504
KT984661.1	460	....	504
GQ422125.1	916	............M..	960
AF531060.1	463	....	507
AF531059.1	463	....	507
AF531055.1	463	....	507
AF531054.1	463	....	507
GQ339856.1	463	....	507
AF531072.1	463	....	507
GQ339862.1	463	....	507
GQ339819.1	463	....	507
GQ339850.1	463	....	507
GQ339842.1	463	....	507
GQ339815.1	463	....	507
AF531070.1	463	....	507
GQ339835.1	463	....	507
GQ339871.1	463	....	507
GQ339861.1	463	....	507
GQ339858.1	463	....	507
GQ339853.1	463	....	507
GQ339846.1	463	....	507
GQ339844.1	463	....	507

GQ339832.1	463	....	507
GQ339831.1	463	....	507
GQ339829.1	463	....	507
GQ339826.1	463	....	507
GQ339817.1	463	....	507
GQ339812.1	463	....	507
GQ339808.1	463	....	507
AF531068.1	463	....	507
AF531067.1	463	....	507
D37821.1	667	....	708
GU055218.1	916	....I.......M..	960
GQ339870.1	463	....	507
GQ339868.1	463	....	507
GQ339866.1	463	....	507
GQ339865.1	463	....	507
GQ339863.1	463	....	507
GQ339859.1	463	....	507
GQ339855.1	463	....	507
GQ339849.1	463	....	507
GQ339848.1	463	....	507
GQ339847.1	463	....	507
GQ339845.1	463	....	507
GQ339841.1	463	....	507
GQ339840.1	463	....	507
GQ339838.1	463	....	507
GQ339836.1	463	....	507
GQ339834.1	463	....	507
GQ339833.1	463	....	507
GQ339828.1	463	....	507
GQ339827.1	463	....	507
GQ339825.1	463	....	507
GQ339823.1	463	....	507
GQ339821.1	463	....	507
GQ339820.1	463	....	507
GQ339810.1	463	....	507
GQ339809.1	463	....	507
AF531075.1	463	....	507
AF531071.1	463	....	507
AF531069.1	463	....	507
GQ339807.1	463	....	507
JF411740.1	463	....	507
GQ339814.1	463	....	507
EF106736.1	991	...........L..	1032
GQ339854.1	463	....	507
GQ339852.1	463	....	507
GQ339816.1	463	....	507
GQ339811.1	463	....	507
D37824.1	664	........H......	708
KT984665.1	460	............M..	504

KM880116.1	586	....	627
EU117991.1	1695	...........L..	1736
KM880119.1	583	............M..	627
KM880120.1	583	............M..	627
GQ339843.1	463	....	507
AF474246.1	1549	....T.......L..	1593
AY600517.1	1548	....T.......L..	1592
AF531074.1	463	............M..	507
AF531073.1	463	............M..	507
GQ339839.1	463	............M..	507
AY713445.1	1663	...I.......L..	1704
U56928.1	1657	...I.......L..	1698
GQ339867.1	463	....I.......L..	507
EF106737.1	952	..R.........T.	993
JF411741.1	463	....I.......M..	507
GQ339860.1	463	....V.......M..	507
DQ407174.1	421	....	459
DQ407189.1	421	....	459
DQ407181.1	421	....	459
DQ407184.1	421	....	459
DQ407173.1	421	....	459
DQ407172.1	421	....	459
DQ407169.1	421	....	459
DQ407188.1	421	....	459
DQ407187.1	421	....	459
DQ407186.1	421	....	459
DQ407182.1	421	....	459
DQ407180.1	421	....	459
DQ407176.1	421	....	459
DQ407171.1	421	....	459
DQ407168.1	421	....	459
DQ407175.1	421	....	459
DQ407185.1	421	....	459
DQ407170.1	421	....	459
DQ407190.1	421	L	459
DQ407179.1	421	L	459
